# Structural Variabilities in β-Lactamase (blaA) of Different Biovars of Yersinia enterocolitica: Implications for β-Lactam Antibiotic and β-Lactamase Inhibitor Susceptibilities

**DOI:** 10.1371/journal.pone.0123564

**Published:** 2015-04-28

**Authors:** Neelja Singhal, Abhishikha Srivastava, Manish Kumar, Jugsharan Singh Virdi

**Affiliations:** 1 Department of Microbiology, University of Delhi South Campus, Benito Juarez Road, New Delhi, India; 2 Department of Biophysics, University of Delhi South Campus, Benito Juarez Road, New Delhi, India; Wake Forest University, UNITED STATES

## Abstract

Yersiniosis caused by *Yersinia enterocolitica* has been reported from all continents. The bacterial species is divided into more than fifty serovars and six biovars *viz*. 1A, 1B, 2, 3, 4 and 5 which differ in geographical distribution, ecological niches and pathogenicity. Most *Y*.*enterocolitica* strains harbor chromosomal genes for two β-lactamases, *bla*A an Ambler class A penicillinase and *bla*B an Ambler class C inducible cephalosporinase. In the present study, susceptibility to b-lactam antibiotics and β-lactamase inhibitor was studied for *Y*. *enterocolitica* strains of biovars 1A, 1B, 2 and 4. We observed that β-lactamases were expressed differentially among strains of different biovars. To understand the molecular mechanisms underlying such differential expression, the sequences of genes and promoters of *bla*A were compared. Also, the variants of blaA present in different biovars were modeled and docked with amoxicillin and clavulanic acid. The mRNA secondary structures of blaA variants were also predicted *in-silico*. Our findings indicated that neither variations in the promoter regions, nor the secondary structures of mRNA contributed to higher/lower expression of blaA in different biovars. Analysis of H-bonding residues of blaA variants with amoxicillin and clavulanic acid revealed that if amino acid residues of a β-lactamase interacting with amoxicillin and the clavulanic acid were similar, clavulanic acid was effective in engaging the enzyme, accounting for a significant reduction in MIC of amoxicillin-clavulanate. This finding might aid in designing better β-lactamase inhibitors with improved efficiencies in future.

## Introduction

*Yersinia enterocolitica*, the causative agent for Yersiniosis has been reported from all continents, but is most common in Europe. It is represented by more than fifty serovars and six biovars *viz*. 1A, 1B, 2, 3, 4 and 5 which differ in their geographical distribution, ecological niche and pathogenic potential [1]. Most *Y*.*enterocolitica* strains harbor chromosomal genes for two β-lactamases—*bla*A, a constitutively expressed Ambler class A penicillinase and *bla*B, an Ambler class C inducible cephalosporinase [2]. Resistance in *Y*.*enterocolitica* against penicillins and cephalosporins is primarily due to blaA which is a constitutively expressed Ambler class-A β-lactamase [3]. As in other enteric bacteria, resistance to β-lactams has become common in *Y*.*enterocolitica*. A very successful strategy to overcome β-lactamase mediated resistance and restore the efficacy of β-lactams has been to use inhibitors of β-lactamases like clavulanic acid, sulbactam and tazobactam. Inhibitors form stable intermediates with β-lactamases, ‘tying up’ the enzymes, while the partner-lactam inhibited the drug target in the bacterial cell *i*.*e* penicillin binding proteins. The β-lactam antibiotic/inhibitor combination, amoxicillin-clavulanate (AMC) is one of the most commonly used antimicrobials, for which an increase in resistance has been noted in recent years due to the acquisition of point mutations in β-lactamases [4].

Various factors might lead to differential β-lactam antibiotic/inhibitor susceptibility in bacterial cells, like point mutations in the β-lactamase gene, modifications in the promoters or regulatory regions of the gene, integration of insertion sequences containing efficient promoters *etc*. Yi et al. [5] reported that when *Burkholderia thailandensis* was grown in the presence of antibiotic, point mutations arose in the coding region of β-lactamases followed by mutation in the promoter region. Sarovich et al. [6] identified two single-nucleotide polymorphisms (SNPs)–one in the coding region near the active site and the other within the promoter region of β-lactamase gene (*bla*) that directly increased ceftazidime hydrolysis by *Burkholderia pseudomallei*.

The objective of the present study was to understand the molecular bases of differential β-lactam antibiotic and β-lactamase inhibitor susceptibility in *Y*. *enterocolitica* strains of different biovars. In pursuance of this, genes, promoters and secondary structures of mRNA of *bla*A of different biovars were analyzed. The three dimensional (3D) structures of blaA were modeled and docked with amoxicillin (AMX) and clavulanic acid to understand the relationship between amino acid substitutions in binding affinities of blaA for β-lactam/ β-lactamase inhibitor. Our findings indicated that variations in the promoter regions and secondary structures of mRNA were not responsible for higher/lower expression of blaA in different biovars. Docking studies revealed that if amino acid residues of a β-lactamase interacting with amoxicillin and the clavulanic acid were similar, clavulanic acid effectively engaged the enzyme, resulting in a significant reduction in MIC of amoxicillin-clavulanate.

## Materials and Methods

### Bacterial strains

Four clinical strains of *Y*. *enterocolitica* representing biovars 1A, 1B, 2 and 4 were examined. The details of the strains *viz*., biotype, serotype, laboratory accession numbers and country of origin are given in Table 1. All strains were maintained on trypticase soy agar at 4°C.

**Table 1:** MIC of β-lactam antibiotics for *Y*. *enterocolitica* biovar 1A, 1B, 2 & 4 strains.Strain designation.

n	Source (country of origin)	Biovar	Serotype	AMX(mg/L)	AMC(mg/L)	FOX(mg/L)	CPD (mg/L)	CTX(mg/L)
Y. e strainC760	Clinical (India)	1A	O:6,30	>256(R)	>256(R)	4(S)	1(S)	0.064(S)
Y. e strain 8081	Clinical (U.S.A)	1B	O:8	48(R)	16(I)	4(S)	4(I)	0.38(S)
Y. e strain W22703	Clinical (Europe)	2	O:9	32(R)	4(S)	1(S)	0.5(S)	0.032(S)
Y. e strain IP26332	Clinical (Europe)	4	O:3	48(R)	6(S)	3(S)	0.75(S)	0.064(S)

AMX, amoxicillin; AMC, co-amoxiclav; FOX, cefoxitin, CPD, cefpodoxime; CTX, cefotaxime

Alphabet in parenthesis shows the drug susceptibility, R, resistant; I, intermediate; S, sensitive

### Determination of the Minimal Inhibitory Concentration (MIC)

MICs of amoxicillin (AMX), amoxicillin-clavulanate (AMX), cefotaxime (CTX), cefoxitin (FOX) and cefpodoxime (CPD) for different strains of *Y*.*enterocolitica* were determined using E-test (bioMerieux Inc., MO, USA). The protocol followed has been described previously [7]. The MICs were interpreted as per the guidelines of Clinical Laboratory Standards Institute [8].

### Preparation of genomic DNA

Bacteria were grown overnight in trypticase soy broth at 28°C. One ml of the bacterial culture was centrifuged at 8, 000 rpm for 10 min and the pellet was used for DNA extraction. The total genomic DNA was prepared using DNeasy Tissue kit (Qiagen, Hilden, Germany) according to the manufacturer’s instructions. Purified DNA was eluted in sterile water and quantitated spectrophotometrically at 260 nm.

### PCR amplification of complete coding sequence (CCDS) of *bla*A gene

PCR amplification of complete coding sequence (CCDS) of *bla*A was carried out using published primers and protocol described earlier [9] using a My Cycler (Bio-Rad, CA, USA).

### Selection of blaA of different biovars for homology modeling and validation of the protein model

The alignment of blaA of different biovars revealed that the amino acid sequence of strains of biovar 2 and 4 were identical. Aminoacid sequence of biovar 1A and biovar 1B strain was different from each other and from that of strains of biovars 2 and 4. Therefore, the amino acid sequence of strain of biovar 1A, biovar 1B and biovars 2 and 4 was selected for modeling and named as blaAx, blaAy and blaAz respectively. Since, the 3D structure of blaA of *Y*.*enterocolitica* is not known; these were predicted by homology modeling. The pair-wise alignment between the target and template sequences was performed with PDB-BLAST. The 3D structures of blaAx, blaAy and blaAz were built using MODELLER 9.12 (http://salilab.org/modeller/). Of the twenty models built for each of the blaAx, blaAy and blaAz, the 3D model with the lowest modeler objective function was selected. The modeled structures were validated by PROCHECK and Verify3D [10–11].

### Molecular docking

The modeled structures of blaAx, blaAy and blaAz were docked with AMX, and clavulanic acid to evaluate the effect of amino acid sequence substitutions on their binding affinity to β-lactam antibiotic AMX and β-lactamase inhibitor clavulanic acid using AutoDock Vina. The binding poses for each enzyme-ligand were determined and different poses were generated based on the total Dock score. The docking parameters and the procedure have been described previously [6]. Hydrogen bonding and hydrophobic interactions in the enzyme-ligand complex were analyzed by PyMOL [12].

### Analysis of mRNA secondary structure

The mRNA secondary structures of blaA variants were predicted using the webserver mfold at default parameters (http://mfold.rna.albany.edu/). The mfold predicts the energetically favorable, optimal secondary structure of RNA based on physical parameters which affect RNA folding like pH, temperature and local biases in RNA.

### PCR amplification of *bla*A gene including the promoter region

PCR amplification of partial CCDS of *bla*A gene along with the promoter region was performed in a My Cycler Thermal Cycler (Bio-Rad, CA, USA) using primers and protocol described previously [7]. The gels were stained with ethidium bromide (0.5μg/ml) and visualized under UV transilluminator.

### Sequencing of CCDS of *bla*A along with its promoter

PCR amplicons representing CCDS and promoters of *bla*A were purified and sequenced following the published protocol [7]. The CCDSs of *bla*A of *Y*.*enterocolitica* were translated into their corresponding amino acid sequences using the software expasy (www.expasy.org/translate). The amino acid sequences of blaA were aligned by Clustal Ώ. (http://www.ebi.uc.ak/clustal Ώ). Since the initial 30 amino acids are signal sequences which are cleaved before the mature enzyme is released in the periplasmic space, these were excluded from comparative studies. The promoter regions of *bla*A were also aligned and compared by clustal Ώ.

## Results and Discussion

The E-test showed that *Y*.*enterocolitica* strains, irrespective of the biovar were sensitive to certain cephalosporins such as cefoxitin, cefpodoxime and cefotaxime. However, these were all resistant to AMX, though the level of resistance differed among strains of different biovars (Table 1). The β-lactamase inhibitor, clavulanic acid reduced the MIC of AMC for biovars 1B, 2 and 4 strains differentially, indicating that blaA was not only heterogeneous, it might also be resistant to inhibitor, as observed in biovar 1A strain. Earlier studies reported that *Y*.*enterocolitica* strains of bioserovars 2/O: 9 were resistant to both ampicillin and AMX but that of 4/O: 3 and 1B/O:8 though resistant to ampicillin were sensitive to AMX [13]. However, we observed that strain of bioserovar 1B/O: 8 though resistant to AMX showed intermediate susceptibility to AMC, while those of bioserovars 2/O: 9 and 4/O: 3 though resistant to AMX were sensitive to AMC.

**Fig 1:**
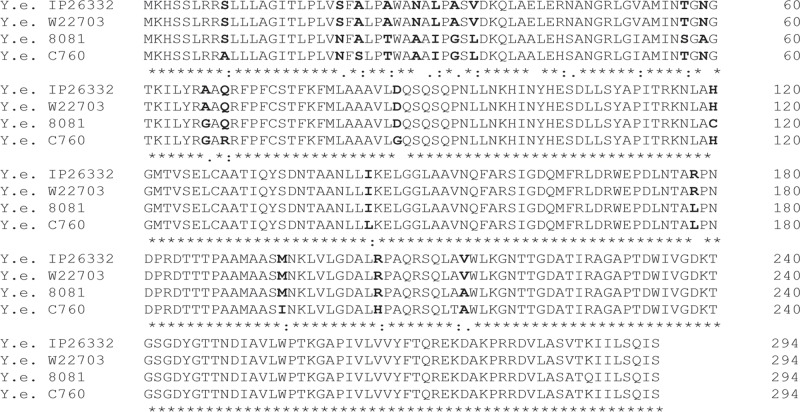
Multiple sequence alignment of the amino acid sequences of blaA from *Y*.*enterocolitica* biovar IA, 1B, 2 and 4 strains. Amino acid substitutions are shown in bold.

The present study aimed at understanding the molecular mechanisms underlying such differential β-lactam antibiotic/inhibitor susceptibilities of *Y*.*enterocolitica* biovars 1A, IB, 2 and 4. To see, if variations in gene sequences of *bla*A were responsible for differential antibiotic/inhibitor susceptibilities, the amino acid sequences of blaA of different biovars were analyzed. The amplification of *bla*A gene using published primers [9] gave the desired product of 896 bp in strains of all biovars. The amino acid sequences of blaA of biovars 1A, 1B, 2 & 4 were quite similar, except for variations in a few amino acids. The sites of variations in blaA of different biovars are shown (Fig 1, Table 2). The amino acid sequence analysis revealed that no amino acid substitution was present in the four significant motifs conserved across class A β-lactamases. In majority of the amino acid substitutions, the chemical nature of the functional groups of the substituent and the substitute were similar thus, affecting the bulkiness of the side chain of the protein without affecting the enzyme-activity. On the other hand, substitution of certain amino acids like glutamate with alanine and arginine with leucine in biovars 1A and 1B might have affected the enzyme activity because the chemical nature of functional groups of the substituent and the substitute were different and the latter lies in the omega-loop region of blaA which constitutes the active site of the enzyme. Similarly, of the three amino acid substitutions observed in biovar 1B, only two might affect the enzyme activity due to difference in the chemical nature of the functional group of the substituent and the substitute.

**Table 2:** Aminoacid substitutions in blaA of *Y*.*enterocolitica* of different biovars.

Sr. No.	Biovar	Amino acid change
	*Y*.*enterocolitica* 1A,1B	L31I
2.	*Y*.*enterocolitica* 1A,1B	A33G
3.	*Y*.*enterocolitica* 1A,1B	V35L
4.	*Y*.*enterocolitica* 1A,1B	E41A
5.	*Y*.*enterocolitica* 1A,1B	R44H
6.	*Y*.*enterocolitica* 1A,1B	N45S
7.	*Y*.*enterocolitica* 1A,1B	V52I
8.	*Y*.*enterocolitica* 1A,1B	A67G
9.	*Y*.*enterocolitica* 1A,1B	R178L
10.	*Y*.*enterocolitica* 1A,1B	V214A
11.	*Y*.*enterocolitica* 1A	Q69R
12.	*Y*.*enterocolitica* 1A	D87G
13.	*Y*.*enterocolitica* 1A	I144L
14.	*Y*.*enterocolitica* 1A	M195I
15.	*Y*.*enterocolitica* 1A	R205H
16.	*Y*.*enterocolitica* 1A	A213T
17.	*Y*.*enterocolitica* 1B	T57S
18.	*Y*.*enterocolitica* 1B	N59A
19.	*Y*.*enterocolitica* 1B	H120C

On the basis of similarities/differences in amino acid sequences, the three types of blaA detected in biovars 1A, 1B, 2 and 4 were named as blaAx, blaAy and blaAz. The 3D modeling of the three blaA types was carried out as substitutions at sites other than active sites of the enzyme, might create local disturbances in the 3D structure and increase/decrease its conformational flexibility, thereby affecting substrate binding. The β-lactamase of *Burkholderia multivorans* (PDB code 3W4Q_A; Uniprot ID A9ANW2) was selected as the template protein for modeling due its high sequence similarity (85%), identity (64%) and low E-value (1e^-115^). PROCHECK validated the modeled blaA types of *Y*.*enterocolitica* showing approx. 90% of the residues in the most favored regions and only 1% in the disallowed regions in the Ramachandran contour plot. Verify3D profiles also showed that 90% region of the protein model of each blaA variant scored > 0.2 which was highly significant.

**Fig 2:**
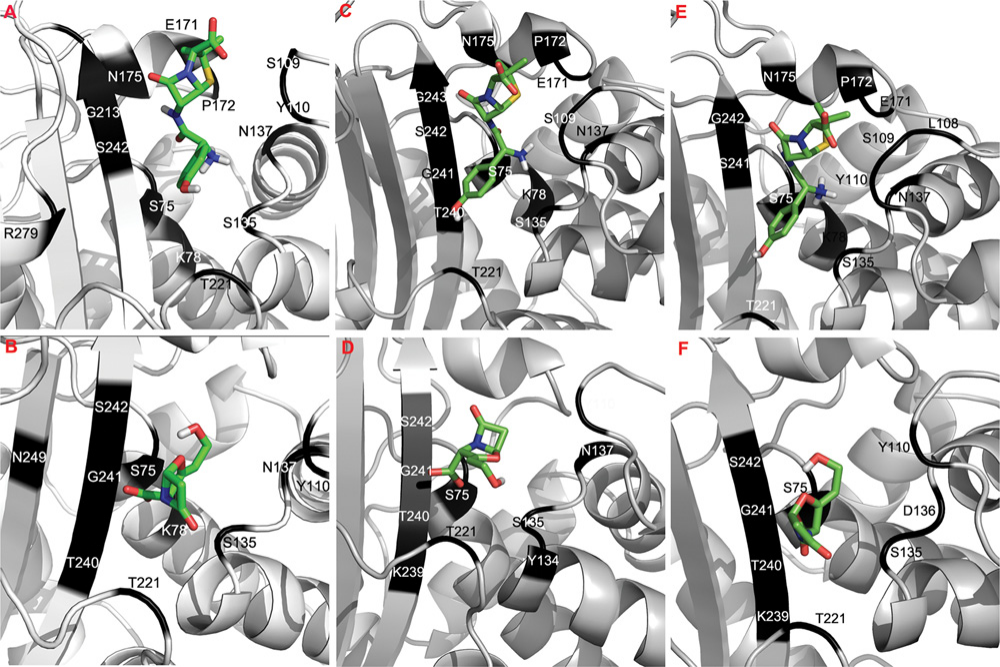
Molecular docking analysis of the blaA variants of *Y*.*enterocolitica*. Molecular interactions of docked blaAx (A & B), blaAy (C & D), blaAz (E & F) with amoxicillin and clavulanic acid respectively. The two antibiotics are represented by stick and the interacting aminoacids are shown in dark color.

*In-silico* docking of the blaA types with amoxicillin and clavulanic acid was carried out. Molecular interactions of the docked complexes of blaAx, blaAy and blaAz with amoxicillin and clavulanic acid are shown in Fig 2. The free energy of binding, estimated inhibition constant, hydrogen bond and hydrophobic interaction of amoxicillin and clavulanic acid with the blaA types were analyzed and the details are given in Table 3. The negative low free energy of binding of docked complexes indicated high affinity of β-lactamases for both amoxicillin and clavulanic acid. It is well known that the inhibition constant (*K*_*i*_) which is equivalent to Michaelis constant (*K*_*m*_) indicates affinity of binding of enzyme-ligand complex. Lower the *Ki*, higher the affinity of β-lactamase for an antibiotic/inhibitor [14]. Based on *Ki*, the blaA types interacted with amoxicillin in the following order: blaAx>blaAy>blaAz, indicating highest affinity of blaAx for amoxicillin, and predicting a greater hydrolysis of amoxicillin by blaAx. The number of residues of blaA involved in H-bonding with amoxicillin was in the following order: blaAx>blaAy>blaAz. More the number of residues involved in hydrogen bonding in an enzyme-ligand complex, greater is the binding affinity of enzyme for a ligand [15].This predicted highest binding affinity of blaAx for amoxicillin and a greater hydrolysis of amoxicillin by blaAx in *Y*.*enterocolitica* biovar 1A. In the present study, we observed that the MIC of amoxicillin for different blaA types was in the following order blaAx>blaAy>blaz. Thus, *in-silico* analysis predicted as well as explained the highest MIC for AMX as observed in case of *Y*.*enterocolitica* biovar 1A.

**Table 3:** Estimated inhibition constants, free energy of binding, H-bond interactions and hydrophobic interactions between amoxicillin and clavulanic acid with blaA types of *Y*.*enterocolitica*.

blaA type	Estimated Inhibition Constant, Ki	Free Energy of Binding (kcal/mol)	Interacting Residues
blaAx_amoxicilin	4.31 uM	-7.32	**H-bonds** (75S, 109S, 135S, 137N, 171E, 242S, 279R); **Hydrophobic interactions** (78K, 110Y, 134Y, 175N, 221T, 240T)
blax_clavulanic acid	392.7 uM	-4.65	**H-bonds** (75S, 135S, 221T, 239K, 242S); **Hydrophobic interactions** (78K, 110Y, 134Y, 137N, 240T, 241G)
blaAy_amoxicillin	5.97 uM	-7.13	**H-bonds** (75S, 135S, 137N, 221T, 240T, 242S); **Hydrophobic interactions** (78K, 109S, 110Y, 171E, 172P, 175N, 241G, 243G)
blaAy_clavulanic acid	2540 uM	-3.54	**H-bonds** (75S, 135S, 221T, 240T, 242S); **Hydrophobic interactions** (110Y, 134 Y, 137N, 239K, 241G)
blaAz_amoxicillin	14.0 uM	-6.62	**H-bonds** (75S, 135S, 137N, 242S); **Hydrophobic interactions** (78K, 108L, 109S, 110Y, 171E, 172P, 175N, 221T, 243G)
blaAz_clavulanic acid	738.69 uM	-4.27	**H-bonds** (75S, 135S, 239 K, 242S); **Hydrophobic interactions** (110Y, 137N, 221T, 240T, 241G)

The number of residues which were involved in H-bond formation with clavulanic acid in different blaA types was in the following order: blaAx = blaAy> blaAz. The *Ki* for blaA types and clavulanic acid was in the following order: blaAy> blaAz>blaAx. However, reduction in MIC brought by the AMX combination was in the order: blaAz>blaAy>blaAx. No co-relation was observed between the *Ki* or the number of H-bonds, and the MIC of different blaA variants. Since the concentration of the inhibitor (clavulanic acid) in each E-test strip was same, the anomalous behavior shown by clavulanic acid might be attributed to the differences in aminoacid sequence of each blaA type which resulted in a different set of residues participating in H-bonding with amoxicillin/clavulanic acid. When the H-bonding residues of blaAy with amoxicillin and clavulanic acid were compared, it was observed that interacting residues were same. Similarly, it was observed that the residues involved in H-bonding with amoxicillin and clavulanic acid in blaAz were similar, except threonine. However, analysis of interacting amino acids of blaAx showed that except for the catalytic serine at positions 75, 135 and 242, amino acid residues that H-bonded with amoxicillin or clavulanic acid were quite different. This implied that clavulanic acid was successful in inhibiting those β-lactamases whose H-bonding residues with clavulanic acid and amoxicillin were similar. This probably might have led to an inhibitor-resistant phenotype observed in blaAx of *Y*.*enterocolitica* biovar 1A.

**Fig 3:**
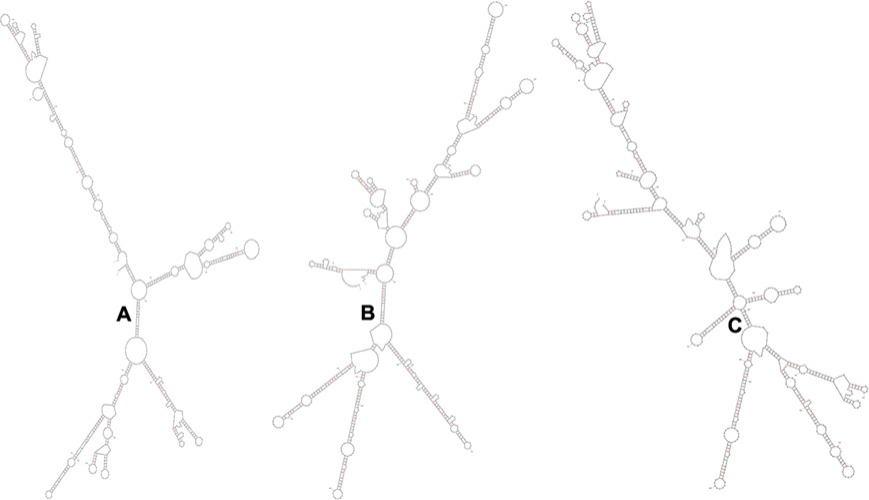
The mRNA secondary structures of blaAx (A), blaAy (B) and blaAz (C) predicted by mfold based on complete coding sequences of different blaA variants of *Y*.*enterocolitica*.

Apart from variations in the amino acids, other confounding factors like secondary structure of mRNA, and/or mutations in the promoters of *bla*A genes might also influence antibiotic/inhibitor susceptibility of blaA types. Therefore the secondary structures of mRNA and promoter sequences of blaA types were also investigated. The mRNA secondary structure directly affects the rate of gene translation, and thus the enzyme activity. The mfold webserver predicted a similar free energy change (ΔG values) and similar mRNA secondary structure for the three blaA types (Fig 3). This implied that the mRNA secondary structure was not responsible for the observed increase in MIC of AMX and/or AMC in *Y*.*enterocolitica* biovar 1A. However, introduction of mutations that would change the secondary structure of the mRNA molecule are required further to validate this finding.

**Fig 4:**
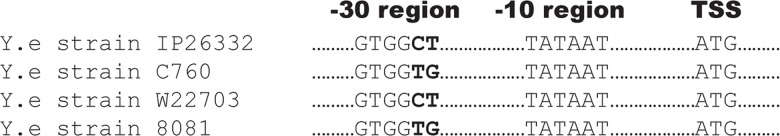
Multiple sequence alignment of the promoter region of *bla*A of *Y*.*enterocolitica* biovar IA, 1B, 2 and 4 strains. The transcription start site (TSS), -10 and -30 regions are shown. The -10 region was conserved but variations were observed in the -30 region.

Mutations and insertions in the promoters of β-lactamase genes have also been reported to be associated with differential expression of β-lactamases [5]. Thus, the nucleotide sequences of promoters of *bla*A of strains of different biovars were analyzed. The sequence of the -10 box of all biovars, TATAAT was identical in all biovars and closely resembled the canonical *E*.*coli* promoter implying that the rate of transcription of *bla*A in *Y*.*enterocolitica* was quite high. It is well known that the sequences in the -35 and -10 regions or sometimes the spacer between these regions affect the transcription efficiency of bacterial promoters. Two single nucleotide substitutions were observed in the -35 region and three in the ribosomal binding region in biovars 1A and 1B (Fig 4). These substitutions might exert lesser influence on enzyme expression, as mutations in the -10 region rather than in -35 regions have been reported to be associated with drug resistance in several members of the family *Enterobacteriaceae* such as *Klebsiella oxytoca* [16]. However, this might be proved by comparison of blaA expression levels in strains of different biovars by quantitative PCR (QPCR). Moreover, these nucleotide substitutions might have not been responsible for higher expression of blaAx which was observed in biovar 1A strain because the same promoter sequence was present in blaAy which showed considerably lower MIC for AMX and AMC.

In conclusion, this study showed that the β-lactam antibiotic/β-lactamase inhibitor susceptibility varied among strains of different biovars. Amino acid sequence comparison indicated that there was limited genetic heterogeneity in blaA of different biovars. Analysis of the secondary structures of mRNA of *bla*A variants and promoter sequences showed that these did not contribute to higher/lower expression of blaA in different biovars. *In-silico* studies though explained the observed high MIC of AMC, but could not explain the high MIC of AMX observed in *Y*.*enterocolitica* biovar 1A. Analysis of H-bonding residues of blaA variants with AMX and clavulanic acid revealed that if the interacting amino acid residues of β-lactamase with AMX and clavulanic acid were similar, the MIC of AMC reduced significantly. This also suggested that if the H-bonding residues of β-lactamase with antibiotic and inhibitor were similar, inhibitor was effective in engaging the enzyme, while the partner lactam antibiotic inhibited the drug target in the bacterial cell, eventually killing the bacteria. This information might serve as an important feature for designing better β-lactamase inhibitors in future. Further experiments on the effect of amino acid mutations on H-binding residues of blaA and their interaction with AMX and clavulanic acid are required to strengthen these findings.
